# Medical Opinion and Movement

**Published:** 1911-04-08

**Authors:** 


### Medical Opinion and Movement.

### Urochromogen in Phthisis.

At a meeting of the Vienna Medical Society, Dr. Moritz Weisz gave the results of his research work on the elimination of urochromogen in pulmonary tuberculosis. According to him the appearance of urochromogen in the urine in the course of the disease is evidence of an active phthisical condition, and its persistence and especially its increase in quantity should be regarded as of serious prognosis, and may be taken as a sign of imminent death. Complications of pulmonary tuberculosis or surgical tuberculosis or influenza may also give rise to the appearance of urochromogen, but the prognostic significance is not then the same. It may also appear after chloroform anæsthesia or tuberculin injections. The elimination of urochromogen is the result of a toxæmia produced by the toxins of the tubercle bacilli. In the majority of cases it is the expression of the passage of the toxins into the blood in massive quantities, and shows either the serious nature of the pulmonary lesions or the feeble powers of resistance of the patient to the bacilli. According to the author, this elimination of urochromogen is intimately connected with the oxidation processes of the cellular protoplasm connected with the defence of the organism to the bacilli and their toxins. The elimination of urochromogen at the commencement of treatment with tuberculin and its subsequent disappearance demonstrates the immunisation of the cells of the organism. On the other hand, its appearance during the treatment shows that the patient is highly sensitive to the tuberculin, or that the dose is too great. The tuberculin treatment may therefore be controlled by repeated examination of the urine for urochromogen.

### Leprosy.

Apart from the gross skeletal lesions produced in the ulcerative mutilating form of leprosy, Dr. Harbitz describes a condition of atrophy which may occur in certain cases of this disease, and which has not hitherto attracted much attention. This atrophy attacks the small bones of the hands and feet, and diminishes in a centripetal direction, so that the carpus and tarsus are less affected. The bones generally preserve their shape, although they become thin, especially in the diaphyses and flat transversely. The most characteristic type is the concentric. In this variety the atrophy attacks chiefly the peripheral extremities of the phalanges, metacarpals, and metatarsals, the bones become smaller and shorter, and assume a conical shape. In this way the heads of the metatarsals and metacarpals may disappear and the phalanges become little ossicles without any relation to each other, and may finally disappear altogether. The changes in the articulations resemble arthritis deformans. The synovial membrane forms vegetations, and a little serous fluid collects in the joints. The articular ends and cartilages atrophy or may become covered with granulations and osteophytes. The bones become extremely porous. The author points out that this atrophic degeneration is evidently of a different origin from the ordinary ulcerative destructive form of the disease, and it is not a sort of leprous osteomyelitis, as it is no way similar to a tubercular or other form of osteomyelitis, and bacilli are seldom found in the bones. The author is led to believe that this osseous atrophy is due to the leprous interstitial nephritis, and points out in support of this theory the similarity between the condition and that occurring in certain nerve lesions, as, for instance, after division of the peripheral nerves. It is further confirmed by the fact that this atrophy of the bones is accompanied by the trophic changes in the skin and by the resemblance between the arthritic changes and those which take place in certain organic nervous diseases, accompanied by trophic degenerations.

### Infantile Cholera.

In the *Bibliotek for Laeger* appears an interesting contribution by Drs. Oerum and Bahr on infantile cholera. Their views are based upon bacteriological research and upon bacteriological and clinical examination of seventy-five cases of this affection. They confirm the general opinion now held that, although improper feeding may be a considerable contributory factor, infection by some pathogenic microbe is largely responsible. Bacteriological examination of a large number of specimens of milk showed the presence of coli-bacilli or para-coli-bacilli, which ordinary boiling fails to destroy. The bacteria collected in the cream are able to resist a high temperature, and afterwards they multiply in the boiled milk more rapidly than in fresh milk. Other sources of infection are " carriers of bacilli " and flies, which have been shown to bear coli-bacilli analogous to those found in the fæces of infantile diarrhœa. Enteritis can be produced experimentally in calves by the injection of para-coli-bacilli, and these bacteria are found present in cases of spontaneous enteritis in these animals. Examination of the stool in infantile cholera usually showed the presence of bacilli belonging to the group of coli-typhoid. Streptococci or the proteus were seldom found. Typical typhoid bacilli were absent. The authors occasionally observed a bacillus resembling the bacillus of Morgan, already described by C. O. Jensen, and also a new form belonging to the para-coli-bacillus group, which they propose to call the pseudo-coli-bacillus. It digests lactose and saccharose, with formation of gas and acids, but not dextrose. It was present in four cases; in two cases it was found also in the urine. Of forty-seven cases in which the fæces and urine were examined at the same time, the same micro-organism was found present in both twenty-five times, a fact which would appear to increase the probability that the micro-organisms were a causal agent of the affection. The authors further confirm the fact that whereas these bacilli of the coli group refractory to Gram are found almost exclusively in infantile cholera, the more usual habitués of the digestive tract, such as the bacillus bifidus communis, tend to disappear.

### Uterine Cancer.

Some interesting figures are given by Dr. Aulhorn in the *Archiv für Gynäkologie* relative to the operation of hysterectomy for uterine cancer. The operations were performed at the Leipzig gynæcological clinic of Dr. Zweifel, and include 420 cases during the last eight years. During this time 641 patients were seen suffering from uterine cancer, but only 420 were considered operable, giving a percentage of 65.5. The percentage of operable cases has, however, gradually risen during this period, so that in 1908 it reached 80 per cent. Of these 420 patients operated upon 56 died, giving a mortality of 13.3 per cent. There were 32 cases of cancer of the uterine body, with two deaths, or a mortality of 6.2 per cent.: 9 of these cases date back more than five years, and are without recurrence. 115 cases of cancer of the cervix operated upon by the method of Wertheim date back more than five years, and of these 11 died immediately, 1 died of some other malady, 1 was lost sight of, 50 have had recurrences, and 52 are alive without recurrences. If these cases are estimated in relation to the total number of cases from which they are derived, including the inoperable cases, they show a cure in 25 per cent. Only 43 patients were operated upon by the vaginal route, with 4 deaths (mortality 9.3 per cent.), but these cases only give 19.4 per cent. cures against 25 per cent. for the abdominal route, so that these statistics are in favour of the abdominal hysterectomy as regards recurrences. In order to diminish the duration of general anæsthesia, Dr. Zweifel commences with spinal anæsthesia. In order to avoid infection of the peritoneal cavity with germs from the uterus or vagina, after freeing the uterus and appendages by the abdominal route he delivers the organs through the vulva.

### Management of Breech Presentation.

In *Surgery, Gynæcology, and Obstetrics*, Dr. Guffey advocates a variation of the usual plan of managing a breech presentation. Instead of allowing the body to be delivered and of then bringing down the arms in the accepted way, he delivers the arm along with the body by passing a hand into the uterus as soon as the feet are born and so capturing the upper limb. He generally chooses the right arm, as pulling it into the left sacro-iliac fossa causes engagement of the head in the right oblique diameter of the pelvis (with the occiput anterior). When the hand has thus been secured and pulled down into the vagina, traction is made both upon it and upon the feet. The obstetrician does not remove his hand from the vagina until the breech is actually born, but grasps the body with it and includes the arm in his grasp. The advantages claimed are that time is saved, as extension of the arm and hitches in the cord are prevented; that there is less danger to the child, owing to this saving of time, and less also to the mother, owing to the lessened liability to lacerations and violence. Again, the hand of the obstetrician, *plus* the child's body, dilate the perineum more than the latter alone, which may save time when the head has to be extracted. In addition, there is less chance of fracture of the humerus or clavicle. The only drawback seems to be the necessity for introducing a hand into the uterus, which is certainly a serious objection to the method.

### A New Intestinal Stimulant.

In the *Buffalo Medical Journal* appears an account of a new German organic product which is highly extolled for constipation, intestinal atony, and all forms of deficient peristalsis. Normal peristalsis is stimulated by a specific cellular product which occurs in substantial quantities in the spleen. This is now isolated and marketed under the name of hormonal. It is sterilised, and its activity is tested by animal experiment. Its action is said to be quite different from that of cathartic purges; even constipation of many years' standing is said to be cured for several months by a single dose injected intra-muscularly. The effect is generally noted about the second or third day after administration. In post-operative intestinal paresis and in peritonitis the results are described as equally striking. For these purposes two forms are made: one containing ¼ per cent. of beta-eucaine hydrochloride, for intramuscular injection in chronic constipation; the other without eucaine for intravenous administration in the treatment of intestinal paresis, etc. Not all cases respond favourably to hormonal, but it has not yet been worked out why some patients are comparatively unaffected while others react speedily and completely.

### More Uses of Hexamethylene-tetramine.

A short time ago attention was drawn in these columns to the use of hexamethylene-tetramine (one of whose trade names is urotropine) for middle-ear suppuration. The suggestion was based originally upon the researches of Custing and Crowe, who proved two or three years ago that the cerebro-spinal fluid has an antiseptic action after this drug has been taken. Several further similar prophylactic and therapeutic uses of this substance have now been suggested and tried, mainly on theoretical grounds in the first instance. Thus in the *Journal of Ophthalmology and Oto-laryngology*, Dr. Dinkelspiel claims that valuable results are obtained in infective eye-lesions, such as indo-cyclitis, hypospyon, and sympathetic ophthalmia. It is especially in the prevention of this latter intractable condition that marked success is claimed. Then again American physicians are using the same drug for anterior poliomyelitis. Dr. Skoog writes of this treatment in the *Journal of the American Medical Association*, and Dr. Clowe in the *New York State Journal of Medicine*. Other authors also report more or less favourably on the method, which consists in the administration of three doses at intervals of one hour, each dose being 2 grains for a child of two years old. No more is then given until the following day, when the same dose is ordered, beginning at the same time as on the previous day. In the *Medizinische Klinik*, Dr. Ibrahim advocates its use in all forms of meningitis in children.

### Cartilage Grafts.

At a recent meeting of the Société de Chirurgie, M. Tuffier gave an account of some interesting experiments he has made in the matter of inter-articular grafts. After making as far back as 1901 an attempt to replace a fragment in a case of fracture, he undertook further experiments on dogs, and has come to the conclusion that it is possible to effect osteo-cartilaginous grafts. Early in 1910 he performed a resection of the elbow for a white swelling in a woman and interposed a cartilaginous graft. At the preceding meeting he showed a young man upon whom he had performed a few months before the classical operation for resection of the elbow-joint for a white swelling, afterwards implanting a cartilaginous graft obtained from a healthy elbow-joint removed for injury. Up to that time he only made use of simple plates of cartilage. The result was good, but it was impossible to show that the cartilaginous tissue in the joint was the same as that placed there when grafting. Early this year he operated on a young man who for some months had been suffering from an infected compound fracture of the elbow. After waiting for the infection to subside he resected a large bony mass, and to the bleeding surface left behind applied the osteo-cartilaginous tissue removed from a leg that had been amputated the same day for injury. He made use of the tibio-tarsal inter-articular cartilage, and finding this of insufficient extent to cover the bone, added more removed from the scaphoid and astragaloid regions. The wound was then closed without drainage being made. Radiography shows that all this cartilage has remained. The elbow articulation now moves through 90°. From these facts he thinks that in such cases it is possible to effect better results than those obtained by a mere interposition of muscle.

### Epilepsy and Constipation.

Professor Bouchard has expressed the opinion that there exists at times a close connection between epileptiform phenomena and loss of proper intestinal function. This view is upheld by Professor Doumer, who publishes in *Le Nord Médical* details of three cases which came under his observation and in which the connection seemed to be established. The first, a child of seven, had suffered from fits for about a year. No history of syphilis could be obtained either in the parents or in the child. Heavy doses of bromides in no way influenced the fits. The child for six months had suffered from marked muco-membranous colitis without this apparently influencing either the number or intensity of the crises. Electrical treatment by abdominal voltaïsation was instituted, the stools became normal after the third daily sitting, the fits became less frequent and less intense, and in three weeks had ceased entirely. They have not returned for the last seven years. The second case, a man of nineteen, had had fits for three years which occurred about once a fortnight. Though denying syphilis he had been treated by mercurial injections for two years without result. Bromides, though influencing, were not able to prevent the fits. When seen by the author he complained of chronic constipation, which necessitated the taking of laxatives. Similar treatment was instituted in his case, and after the twelfth sitting his power of spontaneous defæcation returned. His fits also ceased and for five years have not returned. The third case, a man of twenty-one, had fits since the age of twelve every five or six weeks, and often wet his bed at night. He had had a long course of mercurial and bromide treatment. Though not chronically constipated, he noticed some difficulty in defæcation a day or two before the crises. Electrical treatment was begun in consequence of this statement, and for the past year he has had but two fits. He is now doing regular military service at Lille. In all three cases the author stopped giving bromides, but ordered a diet rich in vegetables, little meat, and no alcohol.

### Intoxication from Burning Magnesium.

When about to take flashlight photographs, photographers are in the habit of making use of burning magnesium to produce a sufficiently powerful artificial light. The combustion of this metal gives rise to a thick white smoke, which is nothing other than magnesia. Prolonged sojourn in a confined space charged with these vapours cannot but be harmful, for in addition to the intestinal irritation which magnesia sets up, it is quite likely that other and graver consequences may result from the fumes. According to *Paris Médical*, Dr. Lahache has traced the origin of a number of cases of ethmoidal caries which have come under his observation to the use of magnesium in this manner.

### Animal Mydriatics.

Some curious results have been obtained by Catapano as the outcome of experiments with extracts of various organs of the body. The adrenals and the hypophysis cerebri, both in aqueous and alcoholic extract, possess a distinct and even intense mydriatic action upon the pupil. Most of the other parenchymatous organs, including the testes, have but a feeble action of this sort; while the extracts of brain and spleen are entirely inert. Muscle extract is feebly mydriatic. The aqueous extract of the thyroid gland is inactive, but the alcoholic solution has a weak pupil-dilating action. Blood serum has no effect of this sort, except in the case of nephritics (this was discovered some time ago by Schurr and Wieser), and hence the author assumes that the active principles must be in the cells of the organs themselves. They are soluble in water and in alcohol; and since the mydriatic results are obtained no matter at what temperature the experiments are initiated, they are probably not due to enzymes. It is thought possible that the curious facts about nephritic patients—whose urine and blood have mydriatic properties—may be due to stimulation of the adjacent adrenals in those with disease of the kidneys; possibly also interference with elimination may have something to do with the question. The original paper appears in the *Berliner Medizin. Wochenschr.* and in abstract in the *Medical Record.*

